# Saunders’ Medical Hand Atlases

**Published:** 1901-07

**Authors:** 


SAUNDERS' MEDICAL HAND ATLASES.—ATLAS AND EPITOME OF LABOR AND OPERATIVE OBSTETRICS. By Dr. O. Shaeffer, of Heidelberg. From the fifth revised German edition. Edited by J. Clifton Edgar, M. D., Professor of Obstetrics and Clinical Midwifery, Cornell University Medical School. With 14 lithographic plates in colors and 139 other illustrations. Philadelphia and London: W. B. Saunders & Co. 1901. Cloth, $2.00 net.

The fifth German edition from which this atlas has been translated without any alteration has been carefully revised and brought well abreast with the best teaching of the day. The text and illustrations cover fully the fields of obstetrics and obstetrical operations, and will materially aid students and practitioners who are not already familiar with these important subjects.

In subdividing the work, the various presentations have been grouped as (1) favorable, (2) relatively favorable, and (3) unfavorable; and the operations as (1) preliminary operations, (2) operations during labor, and (3) operations immediately following labor. By this arrangement the methods in each case are given "in the order of their severity and the danger involved."

